# Clinical performance of machine-learning algorithms to predict intraoperative hypotension: a meta-analysis

**DOI:** 10.1186/s12893-025-03412-8

**Published:** 2025-12-17

**Authors:** Mahdi Faraji, Narges Norouzkhani, Anahid Bagheri Pour, Parnia Ghanad, Mobina bayani, Melika Arab Bafrani, Alaleh Alizadeh, Sina Seyedipour, Niloofar Deravi, Masoud Noroozi, Mohammad Amin Ebrahimi

**Affiliations:** 1 Student research committee, school of medicine, Ardabil University of medical sciences, Ardabil, Iran; 2 Department of Health Information Technology, School of Management and Medical Informatics, Tabriz University of Medical Sciences, Tabriz, Iran; 3 Iran University of Medical Sciences, Tehran, Iran; 4 Student of Medicine, Zhejiang University, Hangzhou, China; 5 Student Research Committee, School of Medicine, Iran University Of Medical Sciences, Tehran, Iran; 6 Students’ Scientific Research Center (SSRC), Tehran University of Medical Sciences, Tehran, Iran; 7 Student Research Committee, Faculty of Medicine, Mashhad Branch, Islamic Azad University, Mashhad, Iran; 8 Jiroft University of Medical Sciences, Jiroft, Iran; 9 Students Research Committee, School of Medicine, Shahid Beheshti University of Medical Sciences, Tehran, Iran; 10 Department of Biomedical Engineering, Faculty of Engineering, University of Isfahan, Isfahan, Iran; 11 Student Research Committee, Sabzevar University of Medical Sciences, Sabzevar, Iran

**Keywords:** Machine learning, Deep learning, Artificial intelligence, Intraoperative hypotension

## Abstract

**Background and aim:**

Intraoperative hypotension is one of the most common surgical adverse effects, often associated with postoperative organ damage and complications. The use of non-invasive methods to predict hypotension during surgery could significantly reduce these complications. Recently developed machine learning algorithms show promise for improving the prediction of hypotension and enhancing detection during surgical procedures. Our meta-analysis aimed to evaluate the clinical performance of machine learning algorithms for predicting intraoperative hypotension.

**Method:**

A systematic search of PubMed, Scopus, Web of Science, and Google Scholar was conducted to retrieve articles published up to August 2024. Two independent researchers screened observational accuracy studies and also identified randomized controlled trials (RCTs) of AI-guided intraoperative management. Observational studies reporting diagnostic/prognostic accuracy metrics were included in the quantitative synthesis. After evaluating the quality of the studies, data extraction and analysis were accomplished.

**Results:**

Five observational studies, including a total of 42,509 participants, were included in this study. Four studies were conducted in South Korea, and one in Taiwan. The machine learning algorithms applied in these studies for electrocardiography, basal systolic, and diastolic blood pressure. These studies assessed model performance based on sensitivity, specificity, and the area under the ROC curve (AUROC). The overall effect size, represented in a forest plot, showed specificity of 0.73 (95% CI: 0.67–0.79), sensitivity of 0.75 (95% CI: 0.71–0.79), and an AUROC of 0.83 (95% CI: 0.77–0.89). In addition, we identified RCTs evaluating AI/HPI-guided intraoperative management, due to heterogeneous endpoints.

**Conclusion:**

Machine learning algorithms demonstrate acceptable performance and advantages in predicting intraoperative hypotension during surgical procedures. The application of these algorithms could be beneficial in future surgeries to reduce complications.

## Introduction

Intraoperative Hypotension (IOH) has become a significant concern worldwide due to its poor prognosis in determining the patient’s status [1]. The IOH affects approximately 29% of patients undergoing non-cardiac surgeries in community anesthesia settings, particularly those receiving general or neuraxial anesthesia. Young individuals and females are especially susceptible [2]. Studies suggest that the complication risk increases with IOH, making timely diagnosis and effective management crucial to achieving favorable outcomes [3].

Although many methods have been proposed—proprietary indices, general machine learning (ML) models, and so on—the comparative evidence on predictive performance is scattered. Additionally, the absolute MAP threshold for IOH may be defined by different studies (absolute MAP thresholds vs. time-under-threshold), as can the prediction horizon (e.g., 5–15 min), input modality (invasive arterial waveforms vs. non-invasive signals), and model families (from logistic regression and gradient boosting to CNN/RNN/attention architectures) [4, 5]. There have been relatively few head-to-head comparisons between modalities. ML-based prediction, leveraging invasive or non-invasive signals, has been proposed to anticipate short-horizon IOH and potentially open a window for earlier hemodynamic intervention [1].

Accordingly, we conducted a systematic review and meta-analysis to quantify the predictive accuracy of ML algorithms for IOH across invasive and non-invasive monitoring. We also explored prespecified sources of heterogeneity, including monitoring modality, IOH definition, prediction horizon, and model family, to clarify where and how ML delivers clinically meaningful discrimination. The existing results suggest that machine learning algorithms perform similarly across both invasive and non-invasive methods, highlighting their potential for broader application in clinical settings [1].

## Methods

In this systematic review and meta-analysis, we investigated the clinical performance of machine learning algorithms for predicting IOH. The study followed the PRISMA (Preferred Reporting Items for Systematic Reviews and Meta-Analyses) guidelines. The research protocol was registered on PROSPERO (CRD42025642751). The keywords used in the search strategy included machine learning, deep learning, artificial intelligence, and hypotension.

### Search strategy

To extract valid and relevant articles, an advanced search strategy was applied across Google Scholar, PubMed/Medline, Web of Science, and Scopus databases. Based on the main Medical Subject Headings (MeSH) related to machine learning and intraoperative hypotension, we filtered titles and abstracts. We applied the search strategy to each database’s query format, without any language or publication-type restrictions (Table 1). To further ensure comprehensive article collection, we also screened previously included studies and systematic reviews for their referenced lists, minimizing the risk of missing relevant studies. Two independent reviewers conducted the screening, and any disagreements between the authors were resolved through discussion.

**Table 1:** Search strategies for PubMed/MEDLINE, scopus databases and Google scholar

Search engine	Search strategy	Search date	Search results
PubMed/MEDLINE	(“Machine Learning“[tiab] OR “Data Mining“[tiab] OR “Neural Networks“[tiab] OR “Deep Learning“[tiab] OR “Artificial Intelligence“[tiab]) AND (Hypotension[tiab]) AND 2000/01/01:2024/07/30[dp]	2024/08/05	1897
Scopus	**(** TITLE-ABS (“Machine Learning” OR “Data Mining” OR “Neural Networks” OR “Deep Learning” OR “Artificial Intelligence”) AND TITLE-ABS (hypotension) AND PUBYEAR > 1999 AND PUBYEAR < 2025 AND NOT PUBDATETXT (“August 2024” OR " September 2024” OR " October 2024” OR " November 2024” OR " December 2024”)**)**	2024/08/05	1838
Google scholar	allintitle: (“Machine learning”) + (“Hypotension”)	2024/08/05	326
Web of Science	(TI=(“Machine Learning” OR “Data Mining” OR “Neural Networks” OR “Deep Learning” OR “Artificial Intelligence”) OR AB=(“Machine Learning” OR “Data Mining” OR “Neural Networks” OR “Deep Learning” OR “Artificial Intelligence”)) AND (TI=(“Hypotension”) OR AB=(“Hypotension”)) AND PY=(2000–2024)	2024/08/05	1639

### Study selection criteria

The following criteria were considered to determine the studies eligible for inclusion in this meta-analysis:

1. Observational studies that developed/validated ML algorithms to predict IOH and reported accuracy metrics (e.g., AUROC, sensitivity, specificity) or provided sufficient data for 2 × 2 extraction and randomized trials evaluating AI- or HPI-guided intraoperative management with quantitative IOH outcomes (e.g., incidence, duration, time under MAP thresholds).
2. Evaluating the clinical performance of the machine learning algorithms in predicting the risk of IOH would be the primary purpose of the study.
3. The study population should consist of patients undergoing surgery for any medical condition.
4. Reporting the definitions of IOH according to the study design.
5. Predefining the threshold levels for mean arterial pressure and hypotension.

Animal studies, studies not investigating hypotension, and studies that did not use machine learning algorithms were excluded because they were not relevant to the inclusion criteria.

### Study quality evaluation and data extraction

To determine study eligibility, the titles and abstracts of articles were independently assessed by two reviewers against the inclusion criteria for this meta-analysis. Studies that did not meet the specified criteria were excluded. Our researchers screened the full texts of the remaining studies, and eligible studies were selected for data extraction. The data extraction process included the following four categories:

1. Study characteristics that contain the authors’ name, type of studies٫, year of publication, and location of the study.
2. Patients’ Specific demographic factors such as age and nationality.
3. Study design characteristics involving the number of patients, sampling method, study period, and the definition of IOH.
4. Relevant outcomes (i.e., proficiency of machine learning algorithms in intraoperative hypotension prediction). For accuracy studies, extracted outcomes included AUROC, sensitivity, and specificity. For RCTs, extracted outcomes included the incidence and/or duration of IOH and time-under-threshold (e.g., MAP < 65 mmHg), when reported.

To assess the quality of case control, cohort, RCTs, and analytical cross-sectional studies, two researchers independently applied the JBI Critical Appraisal Checklist (https://jbi.global/critical-appraisal-tools).

### Statistical analysis

Data analysis was conducted using Stata 18, and the results were reported with 95% confidence intervals (95% CI). The findings were visualized in a forest plot. To assess the heterogeneity among the eligible studies, we used the I^2^ statistic. When significant heterogeneity (I^2^ > 50%) was detected, a random effect model was applied. Additionally, a sensitivity analysis was conducted by sequentially excluding one study at a time and repeating the meta-analysis to ensure the stability of our findings. Finally, potential publication bias was evaluated by visual inspection of funnel plot symmetry and Egger’s regression test.

## Result

### Study selection

**Fig. 1:**
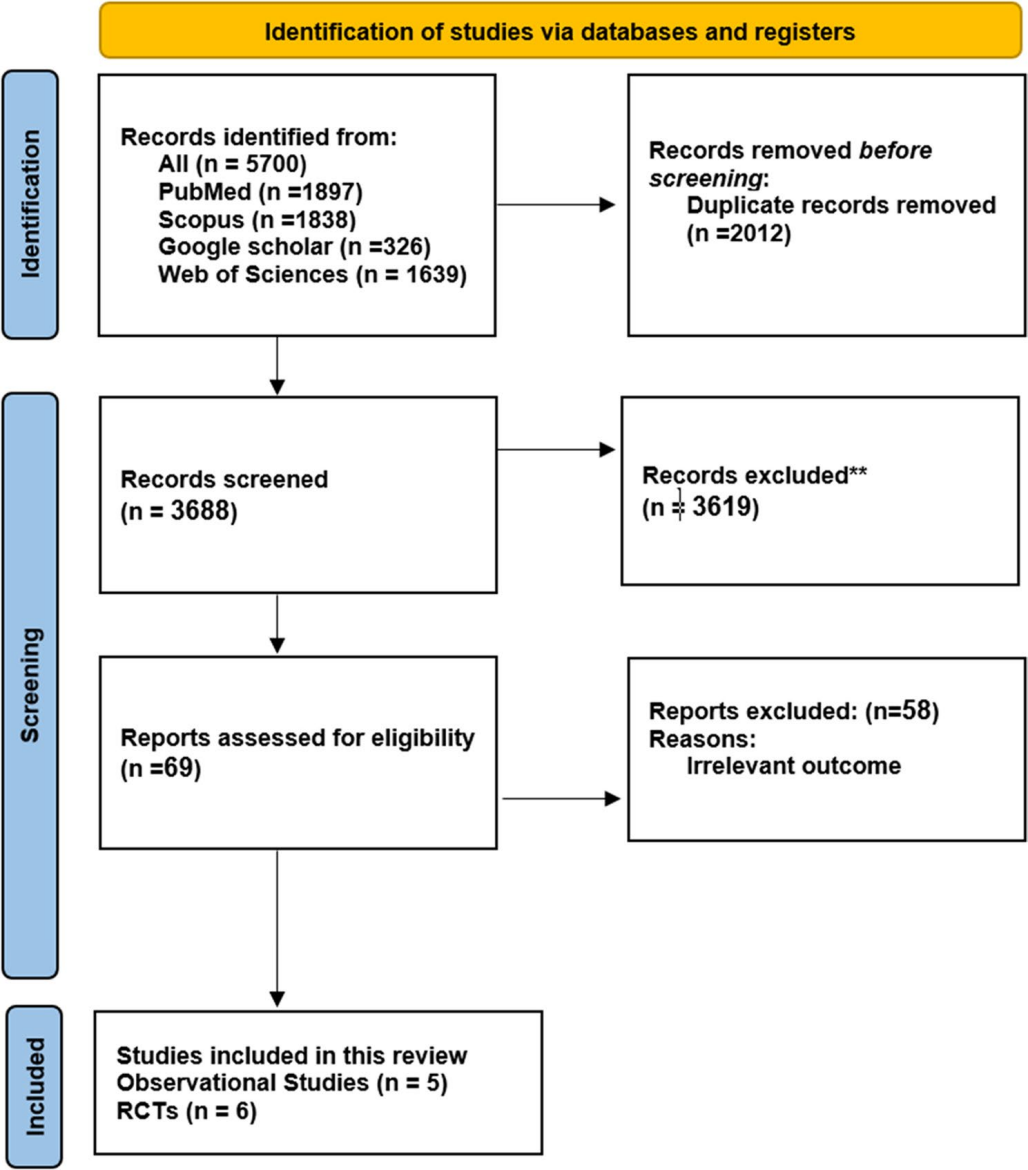
PRISMA diagram

A PRISMA flow diagram (Fig. 1) illustrates the exclusion and screening process. Initial searches across PubMed, Scopus, Google Scholar, and Web of Science identified 5700 potentially relevant articles, of which 2012 were duplicates and were subsequently excluded. After reviewing the titles and abstracts of the remaining 3688 papers, we excluded 963 as unrelated to the review. We then obtained and evaluated 69 full-text articles, excluding 2656 articles that lacked relevant outcomes. Eventually, five studies were deemed eligible and included in this review and meta-analysis.

### Baseline characteristics

This systematic review encompassed five articles involving a total of 42,509 individuals undergoing non-cardiac surgery or receiving spinal anesthesia. The sample sizes ranged from 1,501 to 18,813 participants, and the mean patient age ranged from 31 to 74 years. The included studies were all retrospective observational studies, conducted in South Korea [6–9] and Taiwan [10]. In these five studies, a wide range of machine-learning algorithms, developed and validated using non-invasive data, were utilized. The algorithms primarily relied on basal systolic blood pressure, basal diastolic blood pressure, electrocardiography, and electroencephalography [6, 7, 9, 10]. In some cases, a combination of heterogeneous deep neural networks, especially convolutional neural networks (CNNs) and recurrent neural networks (RNNs), played a crucial role (Table 2).

**Table 2:** Baseline characteristics of included studies

First Author	Year	Country	Participants	Follow up duration	Mean age	Sex	Machine learning models
Jeong et al.[15]	2024	South Korea	4754 of non-cardiac surgery patients	during operation	57 ± 14 years	51% female	algorithm based on blood pressure, electrocardiography, photoplethysmography, capnography and bispectral index
Jeong et al.[15]	2024	South Korea	4754 patients for development, 421 patients for external validation	during operation	-	-	Deep learning with Multi-head Attention and Globally Attentive Locally Recurrent model using 5 non-invasive monitors
Frassanito et al.[16]	2023	Italy	60 women undergoing major gynaecologic oncologic surgery	during operation	Median 59 (IQR 48–69) years	100% female	HPI-guided Goal Directed Therapy (GDT) using invasive arterial pressure waveform
Frassanito et al.[17]	2022	Italy	28 women undergoing major gynaecologic oncologic surgery	during operation	50 ± 10.4 years	100% female	Hypotension Prediction Index (HPI) using non-invasive arterial waveform (ClearSight)
Jeong et al.[18]	2022	South Korea	990 adult patients from VitalDB (ASA I-III)	during operation (MBP recorded every 2 s)	-	-	Deep learning models (DNN + CNN) with stratified loss based on ASA status and permutation (BL, SL, PSL, L2L)
Jo et al.[7]	2022	South Korea	14,140 adult patients undergoing non-cardiac surgery with general anesthesia	At least 20 min during operation	58.8 ± 14.9 years	49.50%	deep neural network trained using ABP, ECG, and EEG waveforms named CNN
Choe et al.[8]	2021	South Korea	554 adult patients (VitalDB) undergoing surgery	during operation	-	-	RNN-based deep learning model (GRU) using ECG, PPG, ABP signals
Choe et al.[8]	2021	Republic of Korea	18,813 subjects undergoing noncardiac surgeries	during operation	58.5 ± 15.3	50.73%	combination of heterogeneous deep neural networks, especially CNNs and RNNs
Lee et al.[9]	2021	Republic of Korea	3301 patients undergoing noncardiac surgeries	during operation			algorithms developed and validated using Bio-signal waveforms acquired from patient monitoring
Lin et al.[10]	2008	Taiwan	1501 patients undergoing surgery under spinal anesthesia	during operation (15 min post spinal anesthesia)	Mean ~ 50 (approximate, from demographics)	54.4% male	Artificial Neural Network (ANN), Simplified ANN, compared to Logistic Regression and clinicians
Lin et al.[10]	2008	Taiwan	1501 patients receiving surgery under spinal anesthesia	during operation	49.5 ± 18.5	45.60%	artificial neural network and Simplified artificial neural network using basal systolic blood pressure (SBP), basal diastolic blood pressure (DBP), and basal heart rate (HR)
Rellum et al. [19]	2023	Netherlands	130	Perioperative (OR phases) + ICU up to 8 h	NR	NR	Hypotension Prediction Index (HPI) — logistic regression-based algorithm embedded in HemoSphere (Edwards Lifesciences)
Lorente et al. [20]	2022	Spain	80	In-hospital until discharge; 30-day mortality assessed	Mean age: ≥65 years	NR	Hypotension Prediction Index (HPI) — ML-based algorithm (arterial waveform analysis) via HemoSphere/Acumen IQ
Tsoumpa et al. [21]	2021	Greece	99 (randomized 100; analyzed 49 HPI, 50 control)	In-hospital postoperative period; complications recorded during stay; no formal 30-day follow-up reported	Median age (IQR): HPI 66 (58–74) years; Control 70 (57–75) years	HPI 53% male (26/49); Control 58% male (29/50)	Hypotension Prediction Index (HPI) — ML-based arterial waveform algorithm (Edwards Lifesciences, EV1000/Acumen platform) integrated into a goal-directed hemodynamic protocol
Feld et al. [22]	2023	USA	1,005 (TBI neurosurgery; train 703/test 302)	Intraoperative only (minute-level; prediction window 5 min)	Median (IQR): 57 (37–73) years	71% Male (714/1,005)	Gradient Boosting Trees (XGBoost) for intraoperative minute-by-minute prediction, Logistic regression (LASSO-selected) comparator

In addition to the five accuracy studies included in the quantitative synthesis, we identified 6 RCTs of AI/HPI-guided intraoperative management (Table 2). These trials varied widely in design, intervention protocols, monitoring modality (invasive vs. non-invasive arterial waveforms), and outcome definitions.

## Meta-analysis

**Fig. 2:**
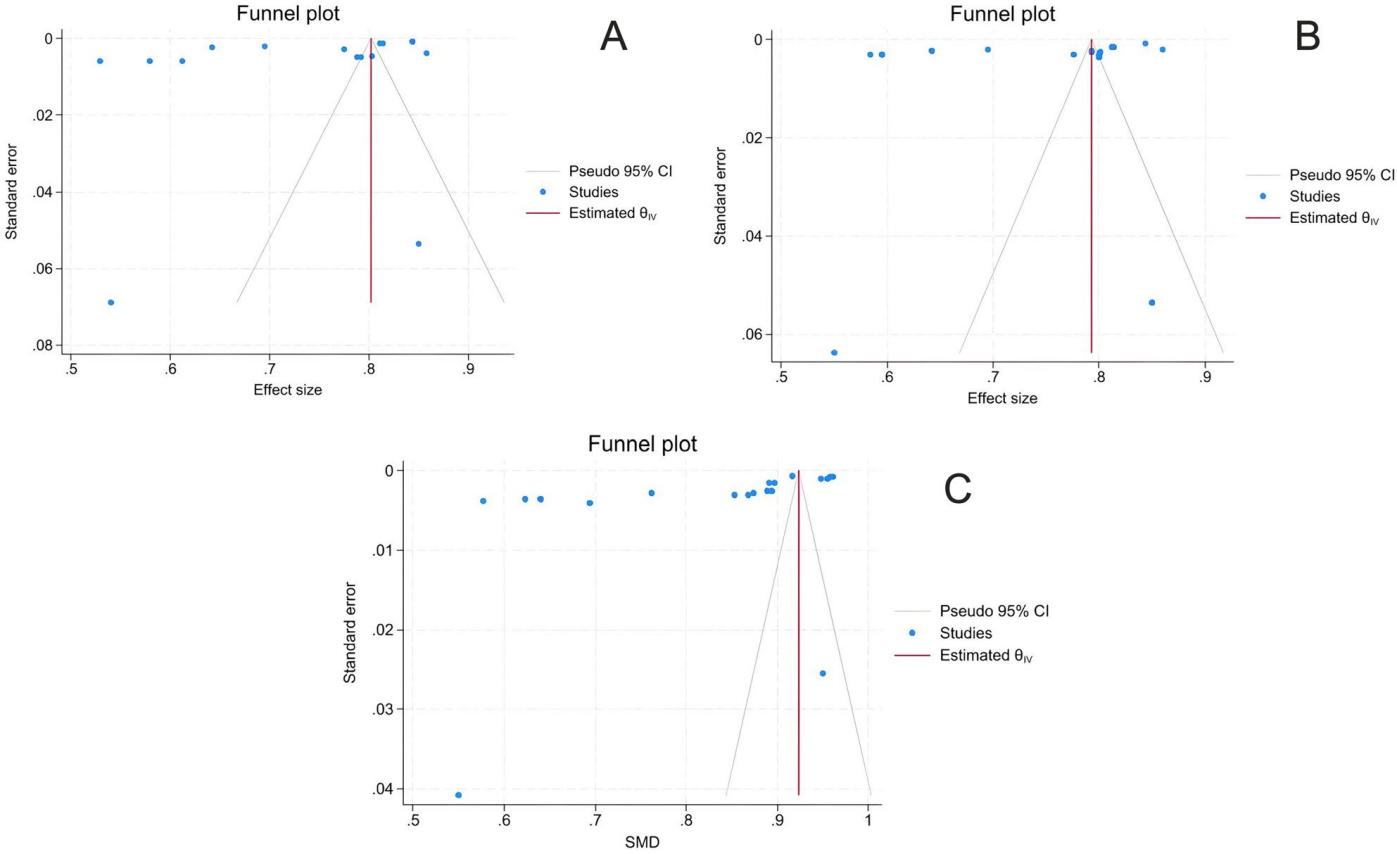
The forest plots showed (**A**) a specificity of 0.73 (95% CI: 0.67 - 0.79), (**B**) a sensitivity of 0.75 (95% CI: 0.71 - 0.79), and (**C**) an AUROC of 0.83 (95% CI: 0.77 - 0.89)

**Fig. 3:**
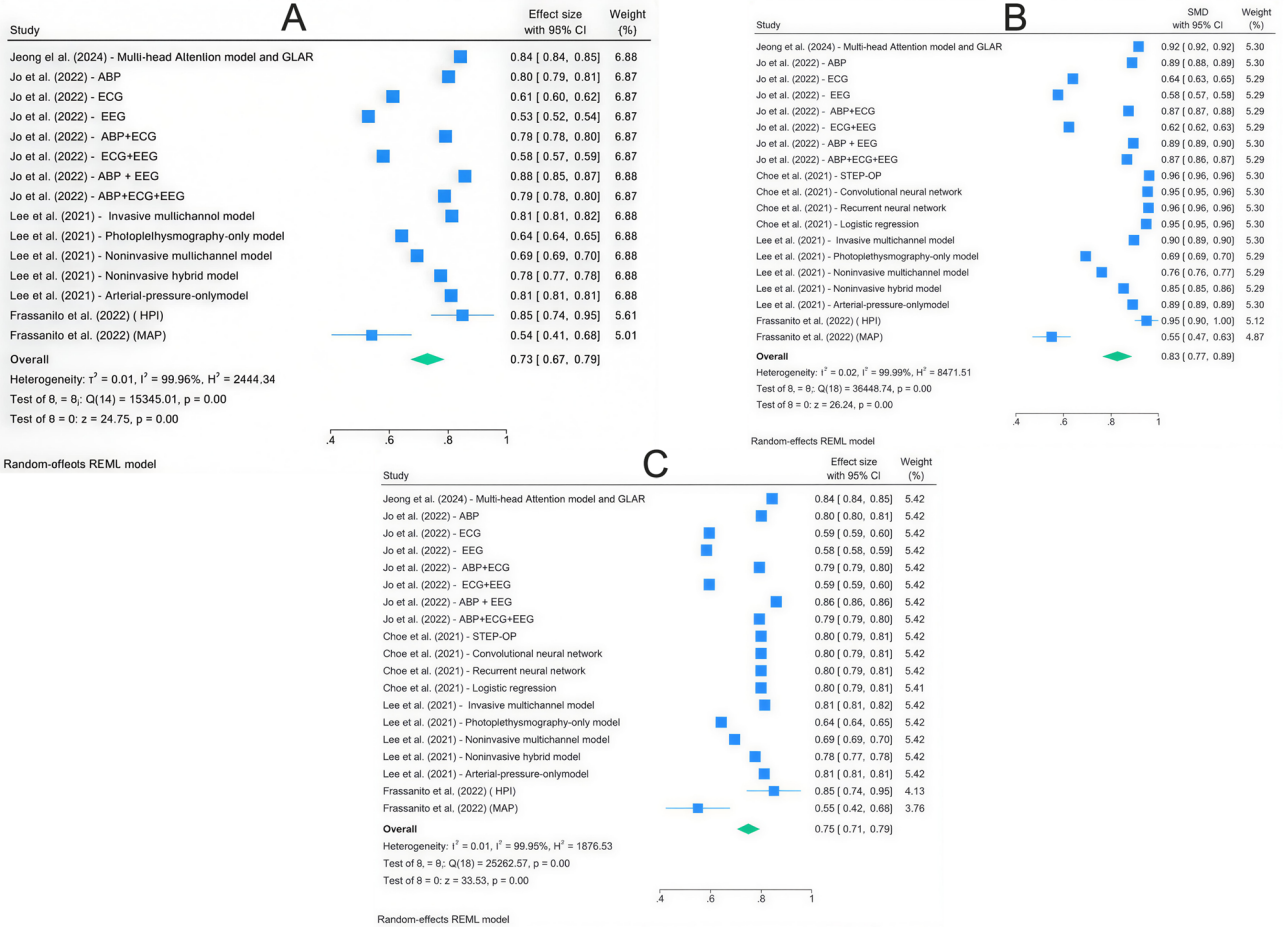
The funnel plots indicated publication bias, with an asymmetrical pattern for (**A**) specificity, (**B**) sensitivity, and (**C**) AUROC

This meta-analysis evaluates the performance of machine-learning algorithms across three metrics: sensitivity, specificity, and AUROC (Fig. 2). The overall effect size, represented in a forest plot, showed specificity of 0.73 (95% CI: 0.67–0.79), sensitivity of 0.75 (95% CI: 0.71–0.79), and an AUROC of 0.83 (95% CI: 0.77–0.89). Substantial heterogeneity was observed among the included studies. All the results were statistically significant, and the funnel plots indicated publication bias, with an asymmetrical pattern (Fig. 3).

### Clinical impact from randomized trials

In the randomized trials identified (Table 2), interventions included HPI-guided care alone or HPI combined with protocolized goal-directed fluid and/or vasopressor strategies. Studies ranged from small single-center trials in specialized procedures to mid-sized pragmatic trials in non-cardiac surgery across multiple countries. Reported endpoints included incidence or duration of intraoperative hypotension and measures such as percentage of time with MAP < 65 mmHg. Trial-level findings varied across studies; detailed characteristics and outcomes are summarized in Table 2.

## Discussion

This meta-analysis assessed the performance of predictive models for IOH using three primary metrics: AUROC, sensitivity, and specificity. The results from all forest plots were consistent and statistically significant, with high heterogeneity indicating substantial variability across studies. Despite this variability, the p-values and random-effect ranges reinforced the robustness of the findings, demonstrating meaningful differences and significance across models.

A recent meta-analysis demonstrated that IOH prediction models, particularly HPI, showed strong performance in predicting hypotensive episodes, which may help reduce the overall duration of hypotension [4]. These findings are consistent with an earlier systematic review by Li et al. [11] that focused solely on RCTs assessing HPI in non-cardiac surgeries. That analysis indicated improvements in several hypotension-related outcomes, including duration, incidence, and percentage of time with MAP < 65 mmHg. Another recent meta-analysis that evaluated a range of AI-supported interventions, including HPI, reported mixed results. While some smaller studies showed promising effects, the largest trial included in the analysis did not find significant differences in hypotension outcomes [12]. Taken together, these findings suggest that the clinical use of HPI remains uncertain. In this context, Michard et al. [13] argued that the predictive power of machine learning algorithms might be overestimated in clinical research, mainly due to selection bias. They also questioned whether these models perform better than simple visual monitoring of arterial pressure. Especially given that some recent prospective interventional trials have suggested that lowering intraoperative hypotension does not necessarily lead to better postoperative outcomes. Although tools like HPI have demonstrated effectiveness in reducing IOH incidence and duration in randomized studies, meta-analyses indicate that other machine learning models, particularly those using CNNs or RNNs, can achieve similar predictive performance [4].

Our results are consistent with previous syntheses demonstrating high specificity/sensitivity of HPI (and other ML) models for IOH prediction, and further refine the scope: HPI for the ability to predict hypotensive episodes has been excellent and may be associated with a decreased duration of hypotension in interventional studies (non-HPI ML models are less specific despite their high AUROCs). Concurrently, randomized evidence of AI-enabled intraoperative management has had mixed effects on pooled hypotension-related outcomes across trials, with a recent meta-analysis demonstrating no significant between-group difference in a composite hypotension outcome (time-weighted area under threshold) [14]. Taken together, these data imply that predictive power alone is necessary but not sufficient; workflow-compatible response bundles and uniform IOH definitions may be required to translate discrimination into clinical benefit.

Randomized trials of AI/HPI-guided management place the bridge between prediction and impact. In different scenarios, “alertness” protocols (fluid, vasopressor, or mixed strategies) guided by invasive or noninvasive hemodynamic monitoring have used an inconsistent definition of hypotension. HPI-guided strategies reduced the burden of IOH in multiple studies and in focused reviews of RCTs—albeit with low-to-moderate certainty of evidence— [15]. In contrast, larger meta-analyses of AA-assisted interventions found no pooled difference for critical hypotension endpoints when trials were combined [14]. That model suggests that fidelity of implementation, including on-time, protocol-driven responses, consensus on the definitions of IOH, and calibrated alert thresholds, is probably required if one desires consistent clinical benefit. It is hoped that future studies will pre-define exchangeable response bundles, minimize risk of contamination, and report standard IOH metrics, allowing for synthesis across studies [12, 13].

### Performance of predictive models

Choe et al. (2021) [8] highlighted the advantages of hybrid deep-learning models, such as CNNs and RNNs, which outperformed logistic regression by utilizing raw arterial waveform data. The ensemble model demonstrated higher AUROC, sensitivity, and specificity, indicating its potential for real-time clinical implementation. These findings emphasize the importance of leveraging raw data for improved performance and offer a pathway for future real-time applications in clinical settings.

Lee et al. (2021) [9] investigated multichannel models that combined invasive and noninvasive signals, achieving superior performance compared to single-channel models. The AUROCs for these multichannel models were consistently higher, supporting the inclusion of diverse bio-signals for enhanced prediction accuracy. Notably, the study emphasized non-invasive methods as a viable alternative in settings where invasive monitoring is not feasible, thereby broadening their clinical applicability in environments where patient safety or resource constraints limit invasive procedures.

Jo et al. (2022) [7] emphasized the importance of combining arterial blood pressure and EEG waveforms, thereby improving predictive performance and calibration for IOH. Their study demonstrated significant AUROC values and meaningful heterogeneity indices, highlighting the value of integrating complementary bio-signal sources to enhance the robustness and accuracy of predictive models. This approach also underscores the role of multimodal data in refining calibration methods for real-time predictions.

Jeong et al. (2024) [6] tested deep-learning algorithms on non-invasive monitors, reporting high AUROC values for internal and external validations (0.917 and 0.833, respectively). These findings highlight the practical utility of attention-based architectures for integrating multi-channel data to predict hypotension. The ability of these models to generalize across datasets, despite differences in monitoring methods and patient populations, demonstrates their robustness and clinical relevance.

Lin et al. (2008) [10] evaluated artificial neural networks (ANNs) and simplified ANNs (SANNs), showing their superior performance over logistic regression and clinician predictions. The streamlined SANNs model, which uses fewer parameters, enhances its applicability in clinical practice, making it a practical option in environments with limited computational resources or data availability. This simplicity, coupled with high predictive accuracy, highlights the potential for widespread adoption of these models.

Randomized evidence of AI/HPI-guided intraoperative management is crucial for bridging the gap between algorithmic performance and clinical impact. In heterogeneous populations, alert-triggered protocols—either based on invasive arterial waveforms or non-invasive signals—have been trialed with diverse definitions of hypotension (absolute MAP thresholds versus time-under-threshold metrics), prediction horizons, and response bundles (fluids, vasopressors, or combination strategies). The contrast and direction of the IOH burden effects have been mixed. They are likely to reflect variation in adherence to implementing the intervention under study (e.g., whether and how strictly a protocolized response is followed and the timing of any action) and characteristics of the population at risk from IOH, as well as the modality of monitoring, frequency of alert, and corresponding clinician workload, in addition to sample size and outcome assessment.

Nevertheless, the causal relationship between hypotension and postoperative complications remains uncertain. Recent randomized trials and meta-analyses have questioned this association. A 2025 meta-analysis of randomized controlled trials (RCTs) revealed no significant variation in postoperative complications between patients maintained at lower versus higher intraoperative mean arterial pressure (MAP) levels [3]. Likewise, another study found no increase in mortality or major complications when permissive hypotension was used in carefully selected patients [4]. Further analysis highlighted the significance of both hypotension severity and duration, noting that prolonged MAP below 65 mmHg was a critical predictor of acute kidney injury [5].

Collectively, these findings show that deriving clinical value from predictive algorithms will not come from a “pure” discrimination game, but from systematizing workflow-congruent response bundles and uniform intervention across hypotension definitions, ensuring that alerts are linked to an actionable threshold. In future trials, protocols should be pre-specified, contamination should be guarded against, and patient-centred outcomes should be powered while relying on standard reporting of common IOH measures to facilitate comparison across trials.

### Strengths of the study

A significant strength of this meta-analysis is its ability to account for high heterogeneity across studies, including differences in IOH definitions, data sources, and clinical settings. By using a random-effects model, the analysis provided reliable pooled estimates despite this variability. All forest plots showed consistent, significant p-values, heterogeneity indices, and random-effect ranges, supporting the strength of the findings and providing a comprehensive view of the predictive value of machine learning in IOH.

### Clinical implications and limitations

The findings of this meta-analysis highlight the potential of deep-learning models to enhance perioperative care. These models enable proactive interventions by accurately predicting hypotension, as demonstrated high AUROC, sensitivity, and specificity metrics. Their ability to integrate multimodal data sources makes them versatile tools across diverse clinical scenarios, thereby improving patient safety and optimizing resource allocation.

However, several limitations must be addressed. The reliance on single-center datasets limits the generalizability of the findings. Additionally, variability in the definitions of hypotension and differences in signal acquisition methods present challenges in standardizing predictive models. While the random-effects model accounted for heterogeneity, further external validation across diverse populations is necessary to ensure broader applicability. We could not meta-analyze RCTs because their outcome metrics and definitions varied substantially and were not directly comparable. As such, RCTs were narratively summarized to contextualize algorithmic accuracy in relation to real-world clinical impact. Future prospective studies should address these limitations and explore integrating these models into routine clinical workflows. Standardizing definitions and validating models in real-world settings will be essential for realizing their full potential in clinical practice.

By addressing these challenges, deep learning models can be effectively integrated into perioperative care, providing a robust tool for predicting IOH and enhancing clinical outcomes.

## Conclusion

This meta-analysis demonstrates the effectiveness of deep learning models in predicting IOH, with significant results across key metrics including AUROC, sensitivity, and specificity. By leveraging hybrid and multichannel approaches, these models achieve high predictive accuracy while addressing a range of clinical scenarios. The use of a random-effects model accounted for heterogeneity, ensuring robust findings and enhancing the reliability of the results. These findings highlight the potential of deep-learning tools to preemptively identify high-risk patients, facilitate timely interventions, and ultimately improve clinical outcomes. Moreover, integrating multimodal data sources enables these models to provide adaptable solutions across diverse clinical settings, including those with limited access to invasive monitoring.

Future research should prioritize external validation to assess generalizability across broader populations and healthcare environments. Additionally, standardizing the definitions of hypotension and addressing methodological inconsistencies will further strengthen the applicability and utility of these predictive tools. By overcoming these challenges, deep-learning algorithms can be seamlessly integrated into routine perioperative workflows, promoting patient safety, reducing adverse events, and optimizing resource allocation across healthcare systems.

## Data Availability

The original data presented in the study are included in the article; the data supporting the results of the present study are only available from the authors upon reasonable request and with permission of the Corresponding authors.
